# Automated Page Turner for Musicians

**DOI:** 10.3389/frai.2020.00057

**Published:** 2020-08-11

**Authors:** André Tabone, Alexandra Bonnici, Stefania Cristina

**Affiliations:** Department of Systems and Control Engineering, University of Malta, Msida, Malta

**Keywords:** page-turning, eye-gaze tracking, Kalman filter, eye-hand span, half-page turns

## Abstract

An increasing number of musicians are opting to use tablet devices instead of traditional print media for their music sheets since the digital medium offers the benefit of storing a lot of music in a compact space. The limited screen size of the tablet devices makes the music difficult to read and musicians often opt to display part of the music page at a time. With fewer music lines on display, the musician will then have to resort to scrolling through the music to read the entire score. This scrolling is annoying since the musicians will need to remove their hands from the instrument to interact with the tablet, causing a break in the music if this is not done quickly enough, or if the tablet is not sufficiently responsive. In this paper, we describe an alternative page turning system which automates the page turning event of the musician. By actively monitoring the musician's on-screen point of regard, the system retains the musician in the loop and thus, the page turns are attuned to the musician's position on the score. By analysing the way the musician's gaze changes between attention to the score and the instrument as well as the way musicians fixate on different parts of the score, we note that musicians often look away from the score and toward their hands, or elsewhere, when playing the instrument. As a result, the eye regions fall outside the field-of-view of the eye-gaze tracker, giving rise to erratic page-turns. To counteract this problem, we create a gaze prediction model that uses Kalman filtering to predict where the musician would be looking on the score. We evaluate our hands-free page turning system using 15 different piano songs containing different levels of difficulty, various repeats, and which also required playing in different registers on the piano, thus, evaluating the applicability of the page-turner under different conditions. Performance of the page-turner was quantified through the number of correct page turns, the number of delayed page turns, and the number of mistaken page turns. Of the 289 page turns involved in the experiment, 98.3% were successfully executed, 1.7% were delayed, while no mistaken page turns were observed.

## 1. Introduction

In this rapidly evolving world, digital media is taking precedence over the physical, printed form for information storage and presentation. Rather than printing books, these are instead being laid out on screens, and whole libraries can now be accessed from one's home or stored within a handheld device. These convenient changes have made it to the world of music. Musical scores are readily available as free, digital documents through digital libraries such as the IMSLP^1^ or as purchasable PDF files from online stores. Digital sheet music offers musicians the advantages of availability and portability, compacting large volumes of works into a single, portable device (Laundry, 2011). Digital sheet music, however, introduces the problem of readability. Traditional, printed music uses the standard A4-size paper, where music players can expect sheet music to have stave heights of 7.5–8.5 mm (Nieweg and Vaught, 2011). The screen-size of regular digital tablets, however, does not permit the display of the entire page while retaining the same stave dimensions. Thus, musicians will either downscale the sheet such that it fits within the space available, or keep the desired size while panning/scrolling to see the entire score (Bell et al., 2005). The latter will require the musician to either pan the score or incur more frequent page turns in comparison to when using a printed score.

Page turns are annoying, requiring the player to momentarily release one hand from the instrument to make the turn. In high-quality music books, editors typeset the music such that the page turn coincides with a natural pause in the music, be it in the form of rests or notes of a longer duration (Laundry, 2011). However, this is not always possible, and musicians develop their particular method to overcome the annoyance of page-turning. Although there are various options to interact with the page on a tablet device, for example, through scrolling or tapping, these actions are not easily controllable when executed at speed. Thus, page-turning on a tablet device is no more comfortable than on print material.

Commercial software and hardware that address this problem exist. These solutions may fall under two categories, namely, manual or fully automated page-turners. Manual page-turning solutions require voluntary user input to trigger a page-turning event. For example, AirTurn^2^ provides a foot pedal system which allows the music player to activate page turns through the use of an external foot-pedal device. While such an approach may be suitable in some cases, some instrumentalists require the use of their feet for their instrument foot pedals (Laundry, 2011). Thus, automated page-turning would be more desirable. Tablet applications such as MobileSheets^3^, SheetMusic^4^, PhonicScore^5^, and ClassicScore^6^ provide such a facility by employing a scrolling score, where the rate of the scroll is determined from the tempo of a pre-recording playback of the music in ClassicScore, which could be adjusted according to some preferred speed in MobileSheets and SheetMusic. Both these options are not ideal since the performer is required to adhere strictly to some specific tempo for the duration of the piece, which, often, results in a performance which is not stylistic. Applications such as PhonicScore allow the scrolling to adjust according to the musician's playing by taking into account real-time audio data. However, these methods are susceptible to background noise, the timbre of the instrument as well as note errors by the performer and are, therefore, not very reliable.

An ideal page-turning system would, therefore, be one which can operate without the use of additional gestures, that is, a system that functions on the already existing interactions between the musician and the score. In this manner, the musician can remain in control over when the page turn occurs while shifting the burden of the actual page turn onto the system controlling the music. Moreover, the page-turning system needs to be robust to errors that may potentially be introduced by the musician.

In our earlier work (Bonnici et al., 2017), we show how eye-gaze tracking can be used to monitor the musician's interaction with the score and thereby create a hands-free page turning system. This system was, however, limited to rigid head and eye movements due to the inherent noise that exists within eye-gaze tracking. In this paper, we extend this work by using a Kalman filter approach to model the musician's gaze interaction and hence provide a robust prediction of the musician's gaze location. We use this information together with a half-page turning system to ensure that the musician will have the current and the subsequent stave present on screen at all times.

## 2. Related Work

**Figure 1:**
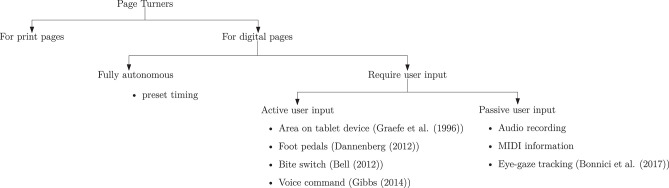
Page turners described in the literature.

Page-turning systems can be broadly categorized into two groups, namely applications for physical, printed books and applications for digital media, as shown in Figure 1. Systems that operate on physical books need to first engage with the top-most printed page. The device needs to lift this page from the remaining pages using mechanisms such as suction tubes, friction wheels, adhesive, or magnetic clips (Wolberg and Schipper, 2012). The page-turner then elevates the single page and transports it, face down, on the other stack of pages. Once turned, the device secures the sheet in place through some restraining mechanism to ensure that the loose sheet does not infringe on any further page-turning actions. Thus, mechanical systems need to balance the speed of turning the page with the relative fragility of the paper so as not to tear the paper (Wolberg and Schipper, 2012). Such mechanisms, therefore, tend to be relatively slow and are most often used in the context of page-turners for people with physical disabilities where the need outweighs speed and efficiency.

Page-turners based on digital media are more common in music applications. The reason for this stems from the increasing availability of tablet devices as well as digital sheet music. As shown in Figure 1, page-turners for digital media can be subdivided into two further categories, namely, those that are fully autonomous and those that depend on some form of user input. Fully autonomous systems rely on preset timing, scrolling through the music sheets at a fixed tempo (Bell et al., 2005). While these systems may allow for manual adjustments of the performing speed at the start of the performance, real-time adaptations to changes in speed are not possible. Thus, these systems are not adequate for musicians. Systems which depend on some user input can, once again, be divided into two categories, those that rely on active user input and those which utilize a passive user-input. Systems which require active user input require some action from the user to activate a page turn. This action can be in the form of tapping a foot pedal (Dannenberg, 2012) or an area on the tablet device (Graefe et al., 1996), bite-switches (Bell, 2012), or voice command triggers (Gibbs, 2014). While some of these techniques may be more effective than others, they still depend on the quick response of the device to the user response. The alternative approach of using passive user-input is, therefore, more attractive for the musician. These approaches involve tracking the musician's progress on the score and use this implicit user-input to determine when to activate a page turn. The tracking can be carried out through audio recordings, through MIDI information obtained from the instrument or by monitoring the users eye-gaze.

### 2.1. Score-Following Systems

When pianists perform a musical piece from a written score, they read the music notation and translate this information into the motor action needed to press the piano keys. The keying-action, in turn, activates the mechanisms that produce an audio signal. Page-turning may, therefore, utilize score-following based on eye-gaze tracking, the keying-action or the audio signal. Eye-gaze trackers typically provide the on-screen (*x, y*) coordinates corresponding to the pianists point-of-regard, and hence, the position on the score from which the pianist is currently playing. The keying action and the audio signal, on the other hand, provide information on the notes played. The data stream obtained from both keying-action and audio signal has a different format to the musical score and, therefore, requires alignment of the data to the score.

In Dorfer et al. (2016), this is carried out by training an end-to-end multimodal convolutional neural network (CNN). The score image *S*_*i*_, consisting of one stave of sheet music, is quantized into overlapping *buckets* *B*_*j*_. Likewise, the spectrogram of the corresponding audio signal is also divided into snippets *E*_*i,j*_ of a fixed length of 12 s. The CNN is trained to match the rightmost onset in the spectrogram *E*_*i,j*_ to the bucket *B*_*j*_ containing the corresponding note *j*. The resulting CNN model is then used to predict the expected location ${\hat{x}}_{j}$ of an audio snippet with a target note *j* in the corresponding sheet music image.

This approach matches the spectrogram within ±1 image bucket in 84% of the test cases. However, the method does not take into account that the music may have repeated patterns which would result in multiple matches between the audio extract and the score. Moreover, the approach also does not take into account the possibility that the performance may deviate from the written score. Such deviations can be intentional, for example, when the musician adds ornaments or chord embellishments not notated in the score. The musician can also introduce temporal changes within the music as a means of expression. These tempo changes would typically affect the estimation of the note onsets (Chen and Jang, 2019). Unintended changes to the performance are also possible, depending on the skill level of the performer. These errors may include incorrect keying of notes, repetitions to correct note errors or note ommissions, resulting in jumps in the performed note sequences (Noto et al., 2019). As a result, the audio extracts may not necessarily have a direct match with the score image.

To correct for the possibility of repeated patterns, Dorfer et al. (2017) introduce temporal information through the use of Dynamic Time Warping (DTW). DTW computes the optimal non-linear alignment between two sequences, using a local cost measure that relates points from the two sequences to each other. In Dorfer et al. (2017), the two sequences comprise of the sheet music and the audio excerpts. A neural network is used to compute a local cost measure between the score sequence and the audio excerpts. The resulting cost matrix is then used by the DTW to align the sheet music and audio excerpts. However, the score-audio alignment is carried out offline. Thus, this approach is not suitable for page-turning applications, which requires real-time alignment of the two.

Chen and Jang (2019) propose an audio-score alignment process based on a similar approach. Note onsets are detected from the audio signal, extracting a feature vector to describe the signal around each onset. Finally, the feature vector is compared to the score using a dynamic programming approach, using a modified constant-Q transform as a measure of similarity. This measure allows for invariance to instrument timbre and overtone interference. To allow for the online score-following, Chen and Jang (2019) then modify the algorithm to reduce the computational time required to align the audio to the score. To achieve this, they assume performance stability and performance continuity. That is, the musician adheres to the tempo marking on the score, and does not introduce sudden tempo changes. The musician is also expected to play the score sequentially and avoid skips or jumps to other sections of the score. These assumptions allow the onset matching algorithm to predict the location of onsets based on the tempo. They also limit the computation of concurrencies to onsets around the previously matched concurrencies. Chen and Jang (2019) achieve a mean latency of 19.2 ms obtained from 10 pre-recorded, human-played, four-part chorales composed by Bach.

While the results obtained in Chen and Jang (2019) do allow for real-time score following, they are based on assumptions of continuity and stability in the performance. These assumptions are valid for performances played when the musical piece has been mastered but do not necessarily hold during practice time when jumps and repetitions can be expected. While jumps and repetitions are difficult to predict through monitoring the audio signal alone, the eye-gaze information can provide invaluable insights on the point-of-regard of the pianist. It is, therefore, possible to deduce the position on the score from which the music is being played. Reading music has commonalities with the reading of linguistic texts, and thus, techniques for gaze tracking in linguistic texts may also apply to musical scores. Unlike linguistic texts, however, music does not have groupings based on fixed words. Instead, groupings are based on pitch structure, temporal structure, articulation, phrasing and orthographic conventions. The visual complexity of the musical score is, therefore, based on the decisions taken on all these levels (Huovinen et al., 2018). When reading, the grouping structures have an essential role in determining the eye movement, defining the duration of the fixations and the landing position of the next fixation.

Fixation points do not necessarily correspond to specific note symbols as long as they lie close enough to the symbol for this to be within the area of vision. This tolerance allows grouped structures, for example, quaver pairs or harmonic chords, to be treated with one single fixation (Puurtinen, 2018). In music reading, fixating on symbols ahead of the current playing position allow the musician to allocate sufficient time to process the symbols while keeping the general rhythmic characteristics of the music. In music reading, this is referred to as reading ahead and results in an eye-hand span. That is, the difference between the notes being played and the fixation point (Rosemann et al., 2016). Any salient difficulties spotted in the score will affect the timing of the saccades launched ahead. Upcoming symbols which appear to be less regular or non-typical will attract first fixations earlier in the musical performance. As a result, the eye-hand span may have local increases due to the musico-visually complex features of the notated score (Huovinen et al., 2018). Moreover, unexpected rhythmic or harmonic changes can locally decrease the eye-hand span (Penttinen et al., 2015; Rosemann et al., 2016).

It is also important to note that although pianists need to look at the score to read the music, they do not do so at all times. In a solo setting, glances to the keyboard are commonplace and help the pianist verify the correct hand position on the keys. Such glances to the keyboard are more common with lower skill level, or when the music leaps through the keyboard registers (Cara, 2018). In ensemble playing, glances at partners are an essential way of communication between the ensemble members (Vandemoortele et al., 2018).

Noto et al. (2019) use Bayesian inference to estimate the pianist's position on the score using both eye-gaze and keying information, integrating the two sources into a single Bayesian inference by using a Gaussian mixture model. The keying and gaze data are modeled by Normal distributions whose parameters are adapted to each subject. The subjects are instructed to play a set extract without stopping or correcting any misplayed notes such that the keying and eye-gaze information can be easily aligned with the ground-truth. An exhaustive dynamic programming search is performed to find the best matching keying pattern from which the average and variance in the most likely matching position is obtained. Likewise, the eye-hand span is assumed to follow a normal distribution with mean (μ_*g*_*x*__, μ_*g*_*y*__) and variance (σ_*g*_*x*__, σ_*g*_*y*__) which are obtained by aligning the gaze data with the expected score position. By learning the eye-hand span distribution, the current gaze point (*g*_*x*_, *g*_*y*_) can be estimated. This estimate is then used in the Bayesian inference model to determine the most likely position for a match between the score and the keying data.

Similarly, Terasaki et al. (2017) also adopt a combined keying and eye-gaze tracking approach. However, Terasaki et al. (2017) use a Hidden Markov Model (HMM) to create a gaze model. The output probability of the HMM follows a normal distribution with the center coordinates of each note as the mean value. The model determines the initial transition probability and the state transition probability by learning the gaze position coordinates (*g*_*x*_, *g*_*y*_) of the gaze when performers are practizing while looking at the musical score. The output probability of the gaze model expresses the gaze likelihood, that is, the probability that the subject is looking at a particular place on the score. This probability score is reflected in the score following by multiplying the cost of the dynamic programming match with the gaze likelihood.

Both these approaches have been evaluated with single-line stave systems, and while Chen and Jang (2019) do take into account the possibility of loss in eye-gaze data, their approach simply waits for the eye-gaze data to become available once more. The two methods also make the general assumption that the eye-gaze will always move ahead. However, in the presence of two stave lines, as typical in piano music, the eye-gaze may also oscillate in the vertical direction. The eye-gaze may also shift backwards when the subject glances at the clef, key-signature and time-signature, particularly if these change within the piece, while at the same time, keying information remains moving forward. Moreover, the eye-hand span may require different local distributions, depending on the characteristics of the piece. Thus, more robust treatment of the eye-gaze information is required.

### 2.2. Displaying the Score

An automated page turning system must also take into account the way the music is displayed on screen and how the page turn is executed. Such a system must take into account the player's experience, allowing the pianist to, not only read the music with ease (Bell et al., 2005; Nieweg and Vaught, 2011) but also to remain well aware of the context of the music they are playing. These considerations will restrict the amount of information that can be presented on the screen while exclude instantaneous jumps between sections of the music (Laundry, 2011). Several options for digital score visualizations have been proposed in the literature. The simplest method offers the presentation of sheet music as a continuous stream, either horizontally with the score scrolling across the width of the screen, or vertically with the score scrolling across the length of the screen. Such digital layouts, however, are not popular with music players since it is easier to lose track of the current position on the score (Bell et al., 2005). Alternative representations, where the score is kept static until a page turn activates overwriting old material with new have been proposed. Here, several visualizations are possible; for example, a two page system may be used with the page turn shifting the whole page to the left, such that the left hand page always displays the current score page to be played while the right hand page displays the next one (Graefe et al., 1996; Blinov, 2007). The screen size of a typical, portable digital tablet, however, does not allow for the display of two pages simultaneously without reducing the page size beyond what can be comfortably read by the music player. Alternative digital music systems which involve displaying a single page make use of the fact that the digital screen may be divided into two parts, allowing for split-page turning whereby, after some time delay, the top part of the page can display new content while the bottom part of the page retains the current content, before this too is updated. In order to indicate the change in content, visualizations such as page peeling, or highlight lines have been used (Bell et al., 2005; Blinov, 2007; Laundry, 2011).

Digital page turning systems must also take into account the display of music with repeated sections, particularly when these sections are long. Since digital displays divide the printed scores into sub-pages for a comfortable fit on the device display space, any such repeat instructions may require going back several pages, aggravating what is already an annoying problem. To resolve the problem, automated page turning can be combined with a system of bookmark annotations to allow the player to go back and forth in the document with greater ease (Jin, 2013). However, instantaneous jumps from page to page in the music are considered distracting to music players (Laundry, 2011). This supports the concept of a flattened score in which all repeats of the musical score are expanded (Jin, 2013). Such a flattened score may be obtained by representing the sheet music using a formal language representation through optical music recognition algorithms, allowing the flattened score to be checked for errors in the interpretation of the repeat instructions (Jin, 2013; Dannenberg et al., 2014; Ringwalt et al., 2015).

## 3. A Kalman Filter Model for Eye-Gaze Page Turning

In this paper, we adopt a Kalman filter approach to create a robust eye-gaze tracking model that can smoothen the noisy eye-gaze data recorded from the eye-gaze tracker while compensating for loss of input due to glances away from the score as well as local variations in the eye-hand span. To model the eye-gaze pattern across the screen, we assume that the score image has been pre-processed using the score processing steps described in Bonnici et al. (2017), that is, the page is sub-divided into sub-pages comprising of two systems, repeats have been flattened and a half-page turn is adopted. We also assume that each system is comprised of two staves as typical of piano music.

### 3.1. Reading Model

**Figure 2:**
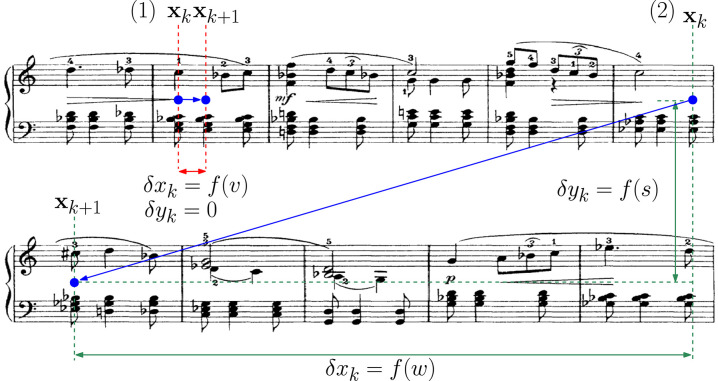
Illustrating a simple reading model. (1) When reading notes within the system, the point-of-regard moves with a horizontal displacement which is a function of the velocity *v* with which the piece is being played. (2) At the end of the system, the point-of-regard moves not only in the horizontal direction, but also in the vertical direction. Here the horizontal displacement is a function of the width *w* of the system, while the vertical displacement is a function of the separation *s* between the two systems.

Music, like text, is read from left to right (Huovinen et al., 2018), such that the current position on the score may be expressed by the linear equation:

(1) $$\begin{array}{l} {\text{x}}_{k+1}={\text{x}}_{k}+\Delta {\text{x}}_{k} \end{array}$$

where ${\text{x}}_{k}={\left(x_{k},y_{k}\right)}^{\prime }$ denotes the current location on the score from which the subject is reading at the instance *k* while $\Delta {\text{x}}_{k}=\;{\left(\delta x_{k},\delta y_{k}\right)}^{\prime }$ denotes the displacement in the reading position. The horizontal component δ*x*_*k*_ of the displacement vector depends on the reading velocity, that is, the local velocity with which the piece is being read which depends on the tempo of the piece as well as the local complexity of the score. Toward the end of the system, however, the horizontal reading position will revert to the start of the next system and is, therefore, a function of the width of the system. Thus, the horizontal displacement may be expressed as:

(2) $$\begin{array}{l} \delta x_{k}=\{\begin{array}{cc} f\left(v\right) & \text{within the same system}\\ f\left(w\right) & \text{at the end of the system} \end{array} \end{array}$$

where *v* is the reading velocity and *w* the width of the system, as illustrated in Figure 2.

Since each system consists of two staves, let us, without loss of generality, assign the vertical component of the reading position to be at the middle of the system as illustrated in Figure 2. While the reading position remains within the same system, this vertical component is expected to remain unchanged. In the transition from one system to the next, this vertical component is expected to shift vertically as a function of the separation between the two systems. Thus, the vertical displacement component δ*y*_*k*_ can be expressed as:

(3) $$\begin{array}{l} \delta y_{k}=\{\begin{array}{cc} 0 & \text{within the same system}\\ f\left(s\right) & \text{at the end of the system} \end{array} \end{array}$$

where *s* is the separation between two systems as illustrated in Figure 2.

An eye-gaze tracker will provide information on the point of regard **g** = (*g*_*x*_, *g*_*y*_) of the subject on the screen. This point of regard corresponds to the subject's current reading position such that the point of regard **g** may be used to adjust and update the reading position predicted through Equation (1). In particular, the point of regard may be used to estimate the local changes in the reading velocity, allowing for updates to both the reading position and the horizontal displacement δ*x*_*k*_. However, from literature on eye-gaze movement during music reading, we know that the eye movement may have variations in the vertical directions corresponding to the subject scanning both staves in the system. The eye-gaze movement will also have horizontal variations around the note being read as the reader shifts their gaze to read upcoming notes. Moreover, glances at keyboards, or partners results in eye-gazes that do not always correspond to the reading location on the score. Thus, the eye-gaze must be considered as a noisy measurement and a method that compensates for noisy data must be adopted.

### 3.2. The Kalman Filter

The discrete time Kalman filter provides such a tool. The Kalman filter assumes that a system is governed by a process modeled by the linear stochastic model given by Equation (4) for which a measurement **z** may be related to the state vector **x** using Equation (5) (Maybeck, 1979)

(4) $$\begin{array}{l} {\text{x}}_{k}=A{\text{x}}_{k-1}+B\text{u}+{\text{w}}_{k-1} \end{array}$$

(5) $$\begin{array}{l} {\text{z}}_{k}=H{\text{x}}_{k}+{\text{v}}_{k} \end{array}$$

where **w** and **v** are random variables representing the process and measurement noise, respectively. These are assumed to be white Gaussian noise processes with zero mean and covariance matrices *Q* and *R*, respectively. Matrix *A* relates the state at the previous time instant *k* − 1 to the state at the current time instant *k*, matrix *B* relates the optional control input **u** to the state and matrix *H* relates the state to the measurement **z**.

The Kalman filter estimates the state inputs in two steps referred to as the prediction step and a correction step, such that feedback from the measurement **z** is used to obtain better estimates of the state vector **x**. The prediction step is is used by the filter to make *a priori* predictions of the state and error covariance using the knowledge gained about the process up to the current time instant. These are denoted as **x**_*k*|*k*−1_ and *P*_*k*|*k*−1_, respectively, and are given by:

(6) $$\begin{array}{l} {\text{x}}_{k|k-1}=A{\text{x}}_{k-1|k-1}+B{\text{u}}_{k-1|k-1} \end{array}$$

(7) $$\begin{array}{l} P_{k|k-1}=AP_{k-1|k-1}A^{\prime }+Q \end{array}$$

The *a priori* state and error covariance estimates are then updated in the correction step which takes into account the most recent measurements obtained at the current time instant. The updated *a posteriori* estimates, denoted by *x*_*k*_|*k* and *P*_*k*|*k*_ are obtained through the correction update step:

(8) $$\begin{array}{l} K_{k}=P_{k|k-1}H^{\prime }{\left(HP_{k|k-1}H^{\prime }+R\right)}^{-1} \end{array}$$

(9) $$\begin{array}{l} x_{k|k}=x_{k|k-1}+K_{k}(z_{k}-Hx_{k|k-1}) \end{array}$$

(10) $$\begin{array}{l} P_{k|k}=\left(I-K_{k}H\right)P_{k|k-1} \end{array}$$

where *K* is a gain matrix which is estimated by the Kalman filter to minimize the *a posteriori* error covariance and weighs the difference between the predicted and actual measurements to update the *a priori* state estiamte **x**_*k*|*k*−1_ to obtain the *a posteriori* state estimate **x**_*k*|*k*_ (Maybeck, 1979).

### 3.3. Application of the Kalman Filter Model for Page Turning Applications

Let us consider the pianist reading music from a single system. If we assume a reading model in which the reading velocity remains constant, then, the process model may be expressed as:

(11) $$\begin{array}{l} x_{k+1}=x_{k}+\delta x_{k} \end{array}$$

(12) $$\begin{array}{l} y_{k+1}=y_{k} \end{array}$$

(13) $$\begin{array}{l} \delta x_{k+1}=\delta x_{k} \end{array}$$

Comparing this model to the process model defined by Equation (4), the reading position within the system may be modeled by a process model with a state vector **x** = (*x, y*, δ*x*)′ such that the matrix *A* is given by:

$$\begin{array}{l} A=\left[\begin{array}{ccc} 1 & 0 & 1\\ 0 & 1 & 0\\ 0 & 0 & 1 \end{array}\right] \end{array}$$

with a zero control input. By considering the eye-gaze position $\text{g}={\left(g_{x},g_{y}\right)}^{\prime }$ as the noisy measurement **z** we can define the matrix *H* as:

$$\begin{array}{l} H=\left[\begin{array}{ccc} 1 & 0 & 0\\ 0 & 1 & 0 \end{array}\right] \end{array}$$

We hypothesize that within the single system the Kalman filter correction step will provide the necessary correction to the state vector **x** to allow for local adjustments in the reading velocity.

Let us now consider the instance when the pianist is transitioning from one system to the next. In section 3.1, we note that this transition requires an additional displacement in the reading position to initialize the reading position to the start of the subsequent system. There are various possibilities to take into account the transition between two systems. For example, if we assume that the transition between the systems is instantaneous, then the additional displacement required can be introduced through the control input **u**. Alternatively, a switching Kalman filter (Murphy, 1998) may be employed to create two reading models, one to model reading within the system and another to model the transition between systems, switching between reading models. However, we hypothesize that for page turning applications, the Kalman filter model will be sufficiently quick in correcting for the reading position when the subject transitions between systems such that no additional inputs or reading models are required. In this manner, we balance accuracy of the reading model with speed and efficiency in the tracking.

### 3.4. Determining the Kalman Filter Parameters

To apply the Kalman filter model, we need to determine the covariance *Q* of the process noise, the covariance *R* of the measurement noise as well as the initial error covariance *P*. To determine estimates for these values, four volunteers were invited to read and play eight set pieces while recording both eye-gaze and keying information. The subjects were asked to play the extracts first as a sight-reading task and then, after allowing a 2-min practice session. Moreover, the extracts were selected such that they contained examples of irregular time and key signatures, varying rhythmic and pitch complexities, and tempo changes. The keying information obtained directly from the MIDI output of the digital piano was synchronized with the score through dynamic time warping. The note onset from the MIDI data was then used to align the eye-gaze information with the keying information and the score. In this manner, we could observe the eye-gaze data under different conditions, allowing for monitoring of variations in the eye-hand span, glances at keyboard and variations on the reading advancements.

From the registration of the MIDI data with the score, we observe that, in general, the pianists position on the score follows the process model described by Equation (13). Variations from this model in the vertical direction exist when the pianist's position on the score shifts from the top to the bottom line of the system. While variations in the horizontal directions are observed mainly due to deviations from the constant tempo model. Using these observations, we empirically determine the initial values for *P*_*k*|*k*−1_ = 0.1*I* where *I* is the identity matrix, and set the process noise covariance to

$$Q_{k}=\left[\begin{array}{ccc} 0.2 & 0 & 0.6\\ 0 & 0.85 & 0\\ 0.6 & 0 & 0.2 \end{array}\right]$$

choosing these values as they best describe the observed variances in the MIDI note onsets. Moreover, from the registration of the point-of-regard and the MIDI data, we observe larger deviations between the point-of-regard and the position on the score. These deviations are due to forward and backward glances as well as vertical oscillations as the pianist reads from both staves of the system. Since these deviations represent the variance that we can expect in the measurement, we empirically set the measurement noise covariance to

$$R=\left[\begin{array}{cc} 5\times 10^{10} & 0\\ 0 & 5\times 10^{5} \end{array}\right]$$

as this best describes the observed variances between the measured point-of-regard and the position on the score.

### 3.5. Loss in the Eye-Gaze Measurement

The discussion thus far assumes that the eye-gaze tracker in use can locate the pianist's eyes at all times. However, from our preliminary study, we note that there are instances when pianists shift their position at the piano, for example, by leaning toward the higher or lower registers of the piano. In doing so, the eyes shift out of the field of view of the eye-gaze tracker, resulting in a loss of eye-gaze measurements. This loss results in measurement data of **z** = 0. While the Kalman filter tolerates noisy data, long instances of erroneous measurements will cause the Kalman filter to diverge, particularly since such losses in the eye-gaze measurements tend to occur over long, consecutive time intervals. Such divergence may lead to accidental page turns which is undesirable.

To compensate for loss in measurement data, we monitor the eye-gaze measurements and in the case of consecutive losses, we interpolate the missing eye-gaze measurements. The interpolation uses the assumed process model such that:

(14) $$\begin{array}{l} {\text{z}}_{1,k}={\text{x}}_{1,k-1|k-1}+{\text{x}}_{3,k-1|k-1} \end{array}$$

(15) $$\begin{array}{l} {\text{z}}_{2,k}={\text{x}}_{2,k-1|k-1} \end{array}$$

When the pianist's eye are once again within the field of view of the tracker, the measurement data will revert to those obtained through the eye-gaze tracker, allowing the Kalman filter to update the state vectors with the new, actual measurement input. Although this approach may introduce some drift, the error due to this drift will not be as large as the divergence caused due to loss in the measurement data.

This approach allows us to use a hybrid model to determine the pianist's location on the score. At instances when measurement data is available, the pianist's position is determined through the Kalman filter eye-tracking model. In the absence of any measurement data, we follow the constant velocity model until sensible measurements are once more obtained from the eye-gaze tracking device. Relying only the interpolation models of Equations (14) and (15) would make the estimation of the pianist's reading position susceptible to the inherent noise of the eye-gaze tracking device as well as variations in the eye-gaze movements as discussed above.

### 3.6. Using the Reading Position to Effect a Page Turn

Page turning is effective if, when the pianist approaches the end of the system on the page, the new system of the subsequent page is already within the pianist's field of view. In this paper, we adopt the half-page turning described in Bonnici et al. (2017), with the score having already been pre-processed to identify the systems, bar-lines and with all repeats flattened. Since the viewing device is intended to be a regular-sized tablet, for readability, each page consists of only two systems displayed at any one time. With two systems per page, half-page turns involve updating one system at a time. Thus, a system *S*_*n*_ will be updated with system *S*_*n*+2_ when the pianist reads from the system *S*_*n*+1_. However, we note that due to looking-ahead habits, toward the end of a system, the pianist may have both systems in focus. Updating a system the instance the gaze is averted to the next system may, therefore, be too distracting for the pianist. For this reason, it is desirable to allow the gaze to settle in the new system before effecting the half-page turn.

**Figure 3:**
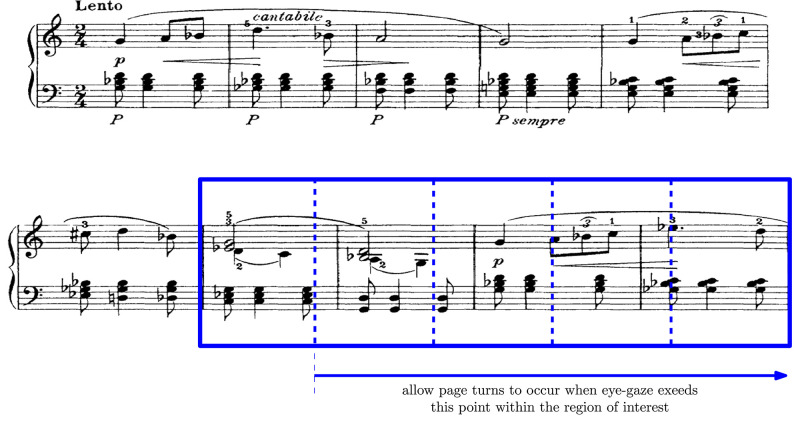
Illustrating the region of interest on the second system. When the eye-gaze position exceeds a set threshold of ⅕ of the region of interest, the page turn can be effected without distracting the pianist.

To achieve this, we create a rectangular area of interest on each new system displayed. This rectangular area of interest spans from the second detected bar-line to the last bar-line of the system as shown in Figure 3. We use this region of interest to accumulate the number of times the pianists gaze falls within the region of interest. By requiring a minimum number of gaze instances within the region of interest, we may ensure that the pianists gaze would have settled on the new system such that effecting the page turn would not be distracting. Empirically, we determine that for pieces played at an average tempo of 120 bmp, we may set the minimum threshold to a fifth of the width of the region of interest. We normalize this threshold with the user-defined average speed of execution of the piece to take into account that faster (slower) average tempo will reduce (increase) the time spent within the region of interest.

## 4. Evaluation Methodology

**Figure 4:**
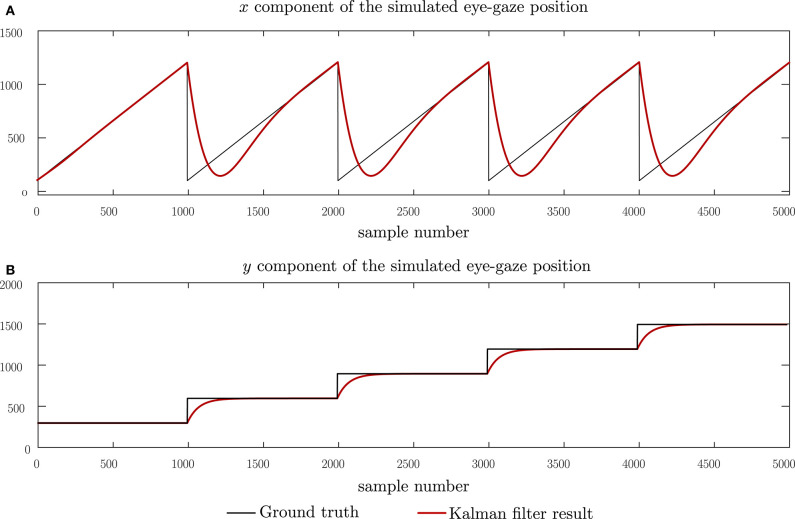
Simulation of eye-gaze measurements while reading five systems and the Kalman filtered result. **(A)** The horizontal component of the eye-gaze movement and **(B)** the vertical component of the eye-gaze movement.

To evaluate the performance of the proposed Kalman filter model and subsequently, the eye-gaze based page-turning, we adopt a two-step evaluation process, using the model first with a set of simulated data, followed by an evaluation with real eye-gaze data. The simulated tests allow us to observe the Kalman filter model with respect to ground truth data and hence, determine the residual error of the Kalman filter model. The simulated ground-truth data was created using the process model described by Equation (1) using a steady tempo of 120 bpm to simulate the reading velocity, a system width of 1,200 pixels, a system height of 200 pixels and a separation of 300 pixels between systems, resulting in an effective page size of 1, 200 × 700 pixels. For simulation purposes, we create a page consisting of five systems as shown in Figure 4. We then introduce perturbations to the ground-truth data to simulate expected characteristics in the real data. We first simulate short-time losses of the eye-gaze measurement data which can be brought about by glances at the keyboard. These are modeled as impulses in both the horizontal and vertical components of the eye-gaze measurements. We then model the noise in the measurement of the point of regard due to micro-saccades in the eye movements as well as noise introduced by the eye-gaze sensor itself. This noise is modeled as additive Gaussian noise, changing the signal-to-noise ratio by varying the variance of the noise distribution. The final simulation attempts to emulate longer losses in the eye-gaze measurements by introducing longer zero-pulses to the ground truth measurement data. This evaluation allows us to compare the effect of the measurement data interpolation on the resulting Kalman filter outcome.

In all these tests, the Kalman filter outputs were expected to follow the ideal input in an over-damped manner due to a tendency of the Kalman filter model to withstand changes in each direction as set in the noise co-variance matrices.

To evaluate the Kalman filter model with real eye-gaze data, we use the SMI RED500 eye-gaze tracker^7^. This eye-gaze tracker uses infrared illumination alongside computer-based image processing to detect the gaze location of the user on a designated area of interest. For optimal conditions of operation, the subject is to sit 60−80 cm away from a 22-inch monitor, where an allowable head box of roughly 40 × 20 cm is formed. Under these conditions, the system offers a binocular tracking with a maximum sampling rate of 500 Hz, contact-free measurement, small automatic head-movement compensation for head movement velocities of up to 50 cm/s by using the corneal reflexes and a typical gaze position accuracy of around 0.4 °. The eye-gaze tracker is connected to a workstation running the iView X™ software. This software facilitates the capturing of eye movements by controlling all the camera equipment and processing all eye and scene video signals captured. This workstation is connected, via Ethernet, to a personal device which hosts our page-turning application. Our application is Matlab-based and communicates to the workstation by using an application programming interface (API) provided by the iView X™ software development kit (SDK). The API allows our page-turning application to control the SMI Red500 and retrieve eye-tracking data.

Using this setup, two tests were carried out. In the first instance, the subject was asked to read and perform 15 piano scores normally, allowing changes in speed within the performance. For this test, 15 different musical pieces were selected such that the pieces exhibited different levels of difficulty and changes in tempo. For these pieces, the performance of the Kalman filter model for page turning applications was evaluated by counting the number of successful page turns, delayed page turns, and advanced page turns. For the purpose of this work, we define successful page turns as those page turns that do not interrupt the flow of music. Delayed page turns are defined as those instances when the pianist has completed the system but the next system is not displayed, introducing a delay in the flow of the music. Likewise, advanced page turns are page turns triggered before the pianist has finished reading the system. These page turns are more disruptive than delayed page turns since they introduce jumps in the music. In the second part of the reading test, we deliberately introduced re-starts and skips in the flow of music to determine whether the Kalman filter model was equally able to retain the successful page turns under large disturbances from the assumed reading model.

## 5. Results

Figure 4 shows the performance of the proposed Kalman filter model under clean, idealized eye-gaze measurements. These measurements will be used as ground-truth when evaluating the performance of the Kalman filter model. From Figure 4, we can observe that, as expected, the Kalman filter acts as an over-damped filter, allowing the system states to reach the desired output. The results shown here demonstrate that the proposed model can follow through changes in reading direction corresponding to shifts in the eye-gaze between different systems. A root-mean-square (RMS) error of 106.0 pixels was observed with this input and this corresponds to a lag between the ideal and predicted states. This lag can be broken down into a lag of 103 pixels in the horizontal direction, equivalent to 8.5% of the page width, and 25 pixels in the vertical direction, equivalent to 3.6% of the page height.

**Figure 5:**
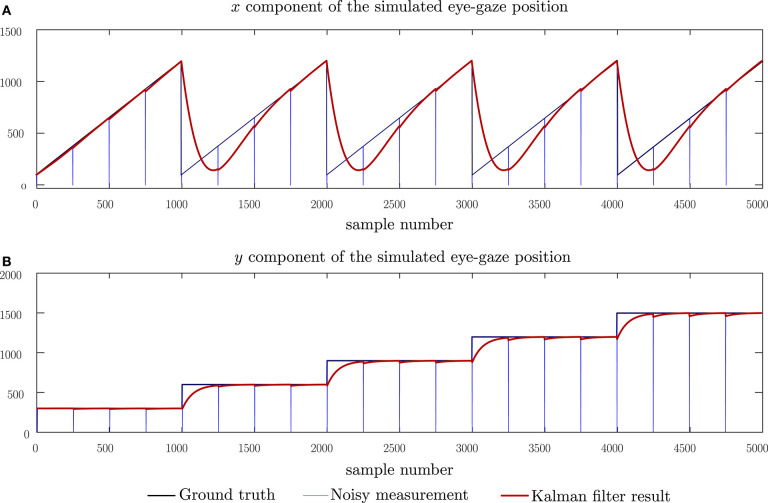
Simulating the loss of eye-gaze data, typical of brief instances when the pianist makes quick glances at the keyboard. **(A)** The horizontal component of the eye-gaze movement and **(B)** the vertical component of the eye-gaze movement.

In Figure 5, we simulate brief losses in the measurement data with impulses inserted at equally spaced intervals along the measurement. We note that the Kalman model filters out these impulses such that the predicted gaze positions lie close to the expected ground truth. An RMS error of 109.9 pixels was observed, which indicates that the difference between the Kalman filter results and the ground truth is mostly due to the lag observed in Figure 4 and that the impulses introduced have little effect on the Kalman filter performance.

**Figure 6:**
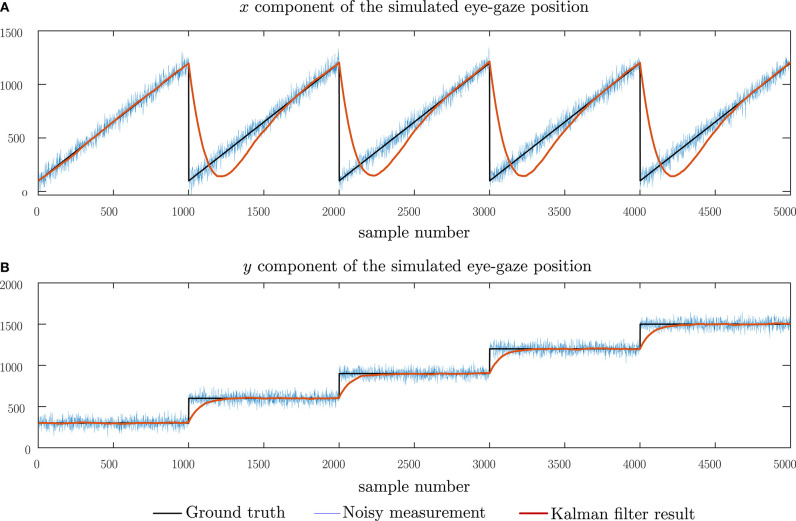
Simulating the performance of the Kalman filter model under noisy measurement data, typical of deviations in eye-gaze due to micro-saccades and noisy sensors. The noise added has a normal distribution with zero mean and a standard deviation of 50 pixels. **(A)** The horizontal component of the eye-gaze movement and **(B)** the vertical component of the eye-gaze movement.

**Figure 7:**
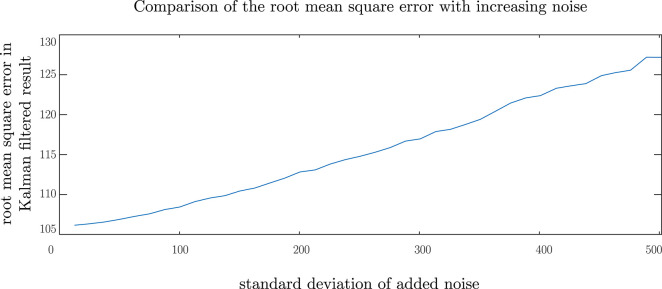
Comparing the performance of the Kalman filter under increasingly noisy data.

**Figure 8:**
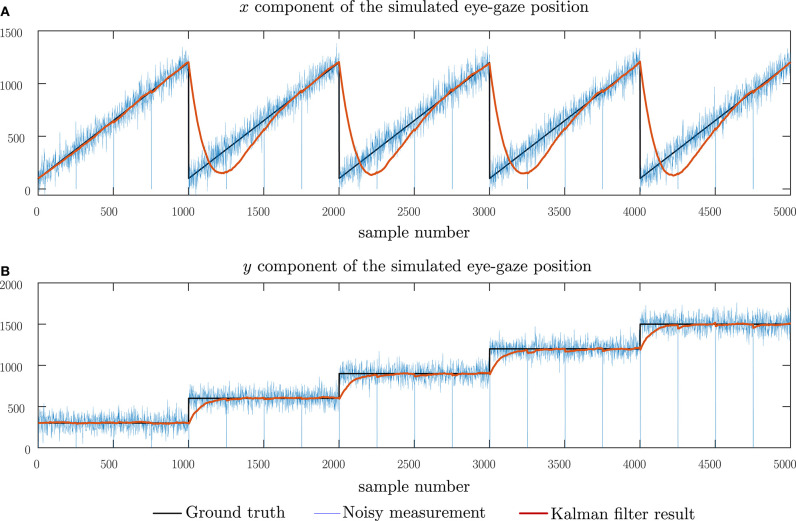
Simulating the performance of the Kalman filter model under combined sensor noise and short instances of measurement data loss. **(A)** The horizontal component of the eye-gaze movement and **(B)** the vertical component of the eye-gaze movement.

Figure 6 shows the performance of the Kalman filter model under the presence of normally distributed noise having zero mean and a standard deviation of 50 pixels. This graph demonstrates how the Kalman filter model adopted compensates for noisy signals and is, therefore, robust to noise in eye-gaze movements due to micro-saccades as well as noise in the sensor itself. For a noise with a standard deviation of 50 pixels, an RMS error of 107.2 pixels was observed. This error is comparable to the error due to the lag introduced by the model. In Figure 7, we show the change in the RMS error with increasing noise up to a standard deviation of 500 pixels. From this graph, we may note that, although the RMS error of the Kalman filter increases, the remaining noise in the filtered data is greatly reduced in comparison with the noise in the measurements. The performance of the Kalman filter model was further observed under combined impulse and normally distributed noise, mimicking instances of noisy sensor and short data losses. The results of this simulation are shown in Figure 8 and, in this case, an RMS error of 111.5 pixels showing that the Kalman filter has the same level of performance under the combined noise models.

**Figure 9:**
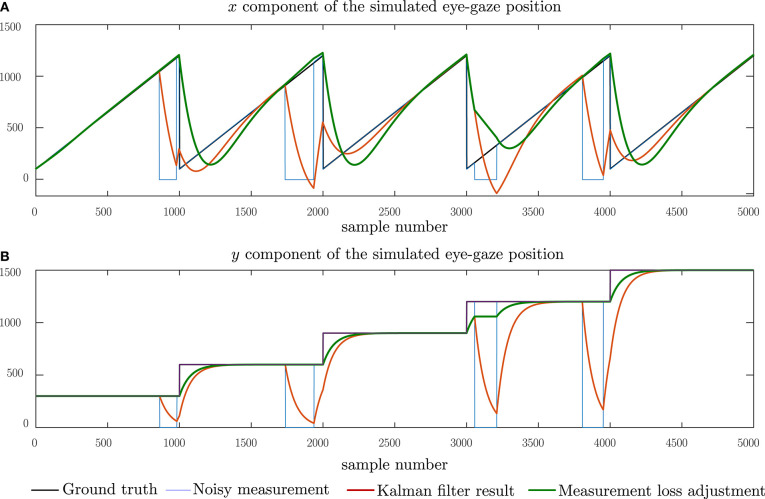
Simulating longer losses in eye-gaze measurements which are typical when the user moves away from the field-of-view of the eye-gaze tracker. The performance of the Kalman filter model (red) can be compared with proposed interpolation of the measurement values to adjust for measurement losses (green). **(A)** The horizontal component of the eye-gaze movement and **(B)** the vertical component of the eye-gaze movement.

In Figure 9, we observe the effect of longer periods of measurement data loss, comparable to instances when the subject's eyes fall outside the field-of-view of the eye-gaze tracker. Here, we compare the performance of the Kalman filter (red) with the same filter model but after performing measurement data interpolation (green). From this result, we may note that loss in the measurement causes the Kalman filter to drift toward the zero level, recovering toward the ground-truth once the measurement data is regained. By applying the measurement data interpolation, the Kalman filter output is being effectively clamped to the constant reading model which not only reduces the drift from the ground truth, but also allows the Kalman filter model to recover from the loss of measurement data more quickly.

**Figure 10:**
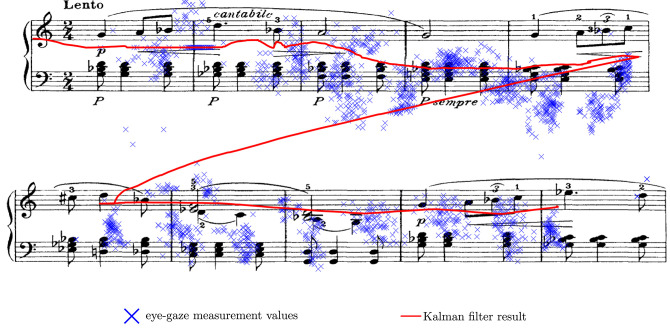
Comparing the eye-gaze measurements obtained from the eye-gaze tracker and the Kalman filter results using the first two lines of Columbine Dances (Martinu) as an example.

**Figure 11:**
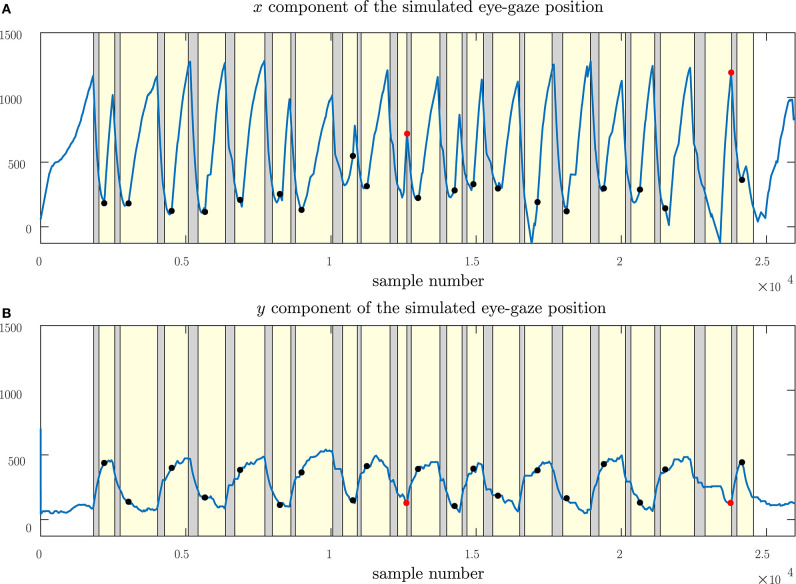
Illustrating the instances where page turns occurred during the execution of Columbine Dances (Martinu). showing **(A)** the horizontal component and **(B)** the vertical component of the eye-gaze movement. Regions highlighted in yellow indicate the position of the region of interest of each system. The occurrence of a successful page turn is marked with black circles while delayed page turns are marked with red circles. Page turns occurring within the region of interest do not cause disturbance in the performance of the piece.

Figure 10 shows the eye-gaze measurements sensed by the eye-gaze tracker while the subject performed the extract. The state-vector from the Kalman filter model is superimposed on this sensed data. Similar to the simulated tests, we can observe that the Kalman filter model reduces the noise in the eye-gaze position estimation, resulting in smoother eye-gaze movements on the score. In Figure 11, we show the Kalman filtered gaze locations and the instances when page turns occur for the entire piece. We superimpose on the graph the region of interest within which, we expect the page turn to occur. Page turns within these regions will ensure that the pianist has the next system in place before reaching the end of the current system. Page turns that occur before the region of interest are likely to disturb the subject by occurring too soon, when the subject is transitioning between systems. On the other hand, page turns that occur too late within the region of interest will delay the page turn, causing the subject to wait for the page turn to occur. In Figure 11, we can observe that of the 21 page turns required to perform Columbine Dances, two of these page turns occurred just at the end of the region of interest, introducing an undesired pause in the music. The remaining 19 page turns occurred within the region of interest and can thus, be considered as successful page turns. Table 1, documents the total number of successful, early and late page turns for the 15 selected pieces. From this table, we note that out of 289 page turns, only 5 page turns were delayed, resulting in a 98.3% successful page turns. The delays observed in the Columbine Dances are due to written tempo change instruction from a slow section to a faster section. In these cases, although the Kalman filter model did adapt to the change in the tempo, the adaptation was not sufficiently quick. The other three delays are mostly due to the score flattening approach adopted from (Bonnici et al., 2017). These pieces had repeat marks within the first half of the system, resulting in very short systems where the image of the written score was cut short to allow for the insertion of the repeated section at the next system. In these cases, our page-turning model required the subject to spend more time within the system before executing a page turn. Adjusting the type-setting of the music through, for example, re-writing the flattened score in MusicXML, would ensure systems of more uniform lengths and hence, eliminate this problem. Nevertheless, the delays incurred were of under 3 s in duration and thus, not unlike the delays experienced when manually adjusting page turns, with the added advantage that the subject can trigger the page turn without needing to remove their hands from the keyboard.

**Table 1:** The performance of the automated page turning on 15 different musical pieces, giving the total number of page turns required as well as the number of successful page turns, the number of late page turns and the number of early page turns as a percentage of the total number of page turns.

**Selected piece**	**Page turns**			
	**Total**	**Successful (%)**	**Late (%)**	**Early (%)**
Columbine Dances, Puppets II, Martinu	21	90.5	9.5	0
Gavotte, Holberg Suite, Grieg	20	100	0	0
Gnosienne No. 1, Satie	15	100	0	0
Children's Corner Suite, Mvt. No. 6, Debussy	18	100	0	0
Impromptu in G flat, Schubert	40	100	0	0
Maple Leaf Rag, Joplin	34	100	0	0
Minuet, from Sonata No. 1, Beethoven	20	95	5	0
Moonshadows On The Mountain, Linn	13	92.3	7.7	0
My Father's Favorite, Doyle	16	100	0	0
Nocturne In C Sharp Minor, Chopin	16	93.8	6.2	0
Papillon Noir, Massenet	15	100	0	0
Prelude In C, from 48 Preludes and Fugues, Bach	10	100	0	0
Song For Sienna, Crain	24	100	0	0
Sundial Dreams, Kern	27	100	0	0
Total	289	98.3	1.7	0

**Figure 12:**
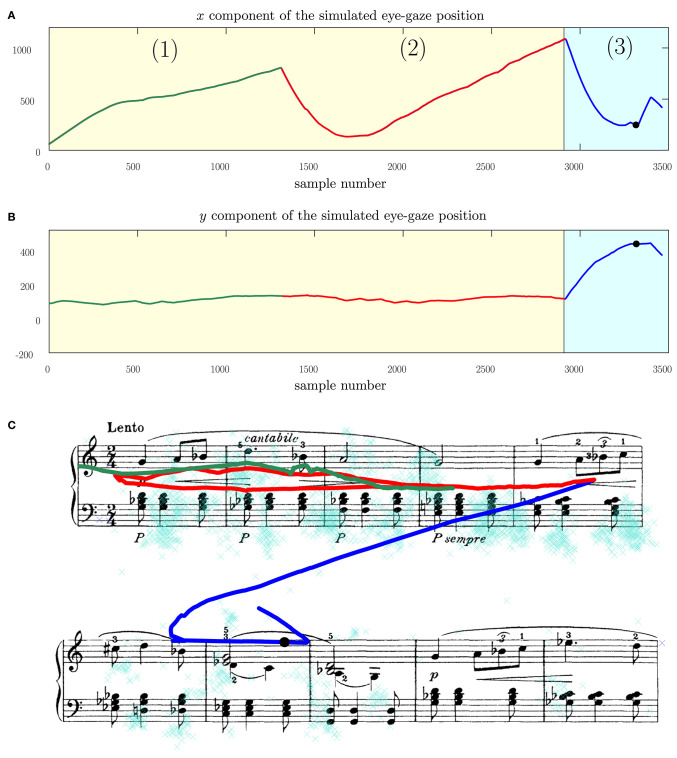
Illustrating the performance of the page-turning under conditions of re-starts and skips, showing **(A)** the horizontal eye-gaze position, **(B)** the vertical eye-gaze position **(C)** the measured and Kalman filtered eye-gaze values on the score. (1) The subject starts by reading the music normally but at (2) stops and restarts the performance from the beginning of the system. The Kalman filter eye-gaze tracking model responds in kind and restarts from the beginning of the system too. The current system remains visible for the subject, causing no interruptions in the flow other than those intentionally introduced by the subject. At (3) the subject proceeds to the next system and the Kalman filter model detects this change. The subject plays the first, second, and third bars of this system, but then skips the fourth bar and goes straight to the fifth bar. The Kalman filter treats such a skip as noise in the measurement model and lags behind. However, the page-turning mechanism can sense that the subject has moved to the second system and can update the first system (not shown here). Thus, when the subject completes the second system, the page is refreshed and can proceed with performing the next system which would be displayed on top.

Figure 12 further shows the performance of the eye-gaze tracking under instances when the subject stops and restarts reading the same section of music. The results shown demonstrate that the Kalman filter model can react to such changes, allowing for the eye-gaze following to function even under changes in the subject's gaze from the expected reading model. Accommodating such changes is necessary as it allows the eye-gaze page-turning to function even under instances of practice time.

For comparison purposes, we performed a subset of the scores presented in Table 1 using the audio-based, page turning function of the PhonicScore App^8^. The music was performed on a Yamaha Clavinova CLP545 digital piano. For purposes of evaluation, the CFX Grand Piano tone was used while the use of pedals was not allowed since any other tone, or the use of pedaling prevented PhonicScore from recognizing the notes being played. The scores were selected on basis of the availability of the music in MIDI and MusicXML file format which are the two file formats recognized by the app. Table 2 gives the number of successful, late and early page turns experienced when using this application. It is important to note that PhonicScore is not restricted to half-page turns and the number of systems presented in a page depends on the density of the music. Overall, the application therefore requires fewer page turns per score. From Table 2, we note that this application has a larger quantity of late and early page turns than our eye-gaze tracking system. Moreover, in all instances, manual intervention was needed to place the cursor position in the correct place on the score. All late and early page turns occurred after the application was unable to match the audio signal with the correct place on the score. In instances of late page turns, the application was unable to pick-up where the user was playing and did not advance at all, whereas in early page turns, the application found matches in places ahead of the user's current position on the score, skipping ahead in the score. These observations further demonstrate the advantages of using eye-gaze tracking for page-turning.

**Table 2:** The performance of the automated page turning using PhonicScore on eight of the pieces given in Table 1.

**Selected piece**	**Page turns**			
	**Total**	**Successful (%)**	**Late (%)**	**Early (%)**
Columbine Dances, Puppets II, Martinu	14	85.7	0	14.3
Gnossienne No. 1, Satie	10	20.0	80.0	0
Children's Corner Suite, Mvt. No. 6, Debussy	12	58.3	0	41.7
Maple Leaf Rag, Joplin	23	56.5	26.1	17.4
Nocturne in C Sharp Minor, Chopin	11	63.6	18.2	18.2
Prelude in C, from 48 Preludes and Fugues, Bach	5	100	0	0
Song For Sienna, Crain	16	50	0	50
Sundial Dreams, Kern	18	61.2	38.8	0
Total	109	61.5	19.3	19.2

*The table presents the total number of page turns required as well as the number of successful page turns, the number of late page turns and the number of early page turns as a percentage of the total number of page turns*.

## 6. Conclusion

In this paper we present an eye-gaze page turning system that allows performers to browse through the music while performing it without lifting the hands from the keyboard. To achieve this page turning system, we describe a simple reading model which describes the way a subject's eye-gaze progresses through the music score when reading and performing the music. This reading model makes assumptions about the reading velocity that are not necessarily strictly observed by the subject. Measurements of the subject's point-of-regard through eye-gaze trackers are therefore used to adjust the position on the score. However, we note that such a sensor introduces measurement noise and thus, we propose a Kalman-filter model to reach a balance between the reading model and the measurement data.

The resulting eye-gaze tracking allows us to create a robust page-turning system which, when paired with a half-page turning display allows constant update of the displayed page such that the subject always has fresh music to play from. The results obtained show that successful page turns occurs in 98.3% of the page turning instances. Furthermore, the page-turning system is robust to instances of re-starts and skips along the system being read.

In our model, we assume that, for the most part, the subject needs to look at the score to read the notes from the score. However, one can envisage instances when the player performs parts of the score from memory. In such instances, our proposed measurement interpolation prevents the Kalman filter model from diverging. Accuracy in the model can be increased if the proposed Kalman filter model is augmented to include a second measurement input, namely, sound measurement. The Kalman filter model would then combine the stochastic nature of the gaze and sound measurements to create a more robust score following.

## Data Availability Statement

The datasets generated for this study will not be made publicly available The eye-gaze data required for the purpose of this study was not recorded during the study. The musical sheets used can be provided, but these are sheets which are readily and freely available from various music libraries.

## Ethics Statement

The studies involving human participants were reviewed and approved by University of Malta. Written informed consent for participation was not required for this study in accordance with the national legislation and the institutional requirements.

## Author Contributions

AT was the primary contributor of the algorithms required to perform this research and carried out this research under the tutorship and supervision of both AB and SC.

## Conflict of Interest

The authors declare that the research was conducted in the absence of any commercial or financial relationships that could be construed as a potential conflict of interest.

## Footnotes

^1^https://imslp.org/

^2^http://www.airturn.com/

^3^http://www.zubersoft.com/mobilesheets

^4^http://www.musicnotes.com/apps/

^5^http://phonicscore.com/

^6^http://blog.naver.com/earthcores

^7^https://imotions.com/hardware/smi-red500/

^8^http://phonicscore.com/
